# Objective Evaluation of Active Interactions between the Operator and Display Screen Equipment Using an Innovative Acquisition System

**DOI:** 10.3390/bioengineering10060686

**Published:** 2023-06-04

**Authors:** Giuseppe De Palma, Emma Sala, Sofia Rubino, Simone Dalola, Marco Ferrari, Daniele Marioli, Pietro Apostoli, Cesare Tomasi, Francesca Righetti, Flavia Mattioli, Vittorio Ferrari

**Affiliations:** 1 Unit of Occupational Health, Hygiene, Toxicology and Prevention, University Hospital ASST Spedali Civili di Brescia, 25123 Brescia, Italy; emma.sala@unibs.it; 2 Unit of Occupational Health and Industrial Hygiene, Department of Medical Surgical Specialties, Radiological Sciences and Public Health, University of Brescia, 25123 Brescia, Italy; sofyrubino@gmail.com (S.R.); pietro.apostoli@unibs.it (P.A.); cesare.tomasi@unibs.it (C.T.); 3 Department of Information Engineering, University of Brescia, 25123 Brescia, Italy; simone.dalola@unibs.it (S.D.); marco.ferrari@unibs.it (M.F.); daniele.marioli@unibs.it (D.M.); vittorio.ferrari@unibs.it (V.F.); 4 Neuropsychology Unit, University Hospital ASST Spedali Civili di Brescia, 25123 Brescia, Italy; frarighetti@gmail.com (F.R.); flaviacaterina.mattioli@gmail.com (F.M.)

**Keywords:** display screen equipment (DSE), video terminal unit (VDU), screen fixation, occupational health, objective measure, monitoring system

## Abstract

The occupational risk of operators using display screen equipment (DSE) is usually evaluated according to the extent of time spent in active operator–DSE interactions. Risk assessment is based on activity data collected through questionnaires. We evaluated an original and innovative system that can objectively assess active operator–DSE interactions by collecting electrical impulses generated by the activation of mouse, keyboard and a camera that collects attentive eye-screen fixation. The main aim of this study was to evaluate the system’s performance on an employee sample involved in the task of active reading and copying at a DSE workstation connected to the system. In the context of mandatory health surveillance at work, we enrolled 38 DSE operators with normal neuropsychological and eye assessments who were required to complete two predefined reading and writing tasks. The obtained results show that the system is able to collect activity data derived from operator–DSE interactions through screen fixation, keyboard tapping and mouse handling. In the copying task, the session duration as recorded by the system was highly related to the screen fixation time. In the copying task, mouse and keyboard activities were more strongly related to session duration than screen fixation. For the copying task, it was also possible to obtain individual profiles of operator–DSE interactions while performing the same standardized tasks. Collected data can allow an objective evaluation of active time spent by DSE operators at their workstations, thus allowing a more accurate occupational health risk assessment and management. Prospective analysis of individual operator–DSE interaction profiles can favor the setup of targeted preventive and organizational interventions from an of even wider worker wellbeing perspective.

## 1. Introduction

Digital technologies are omnipresent in every context of our lives, but prolonged use of digital devices can lead to health disorders, and the potential influence of screen time on health may vary by social context [1,2,3]. In occupational settings, eye fatigue may occur while using display screen equipment (DSEs), possibly giving rise to asthenopia, with symptoms including dry eye disease, diplopy, ocular discomfort, headache, red eyes, sensitivity to light, and blurry vision, the so-called computer vision syndrome (CVS) [4]. Rios et al. published a study with the main aim of investigating which risk factor could have more influence in developing CVS and elaborating an algorithm to predict its development [5]. In the literature, different interventions (i.e., multifocal lenses, blue light blocking, oral berry extract supplementation, omega-3 fatty acid and oral carotenoid supplementation, eye drops, etc.) have been described to prevent and treat CVS symptoms, but scientific evidence is inconclusive [6].

Incorrect and prolonged static postures may contribute to biomechanical overload to the musculoskeletal system, leading to neck, shoulder, and low back pain, and performing word processing activities (typing at the keyboard) for more than 4 h total per day may expose the wrist and hand to biomechanical overload [7,8,9]. The prevalence of DSE operators that suffer from musculoskeletal disorders (MSDs) ranges from 20 to 60% [10]. A recent review [11] showed that posture is a moderate independent risk factor for MSDs among DSE operators. It appears that lowering the keyboard height to or below elbow height and resting the arms on the desk surface or chair arms reduce the risk of neck and shoulder MSDs. On the other hand, daily or weekly hours of DSE use are more consistently associated with hand and arm MSDs. In addition, mental fatigue and noise in open space offices can worsen preexisting disease and can contribute to work-related stress [12,13].

Prevention of DSE-related disorders at work requires a limitation of screen fixation times, as well as the setting ergonomically appropriate DSE workstations. Technical standards, such as UNI and UNI EN ISO [14,15,16,17], are useful for such purposes.

In Italy, the transposition of the EU Council Directive 90/270 of 29 May 1990, poses the health risk threshold for DSE operators at 20 h of active operator–DSE interaction per week [18]. At-risk DSE operators are thus classified according to the time of active operator–DSE interaction, often referred to questionnaires, compiled by the workers themselves or by their department heads.

Two studies published in 2007 and 2012 described a tool to objectively collect operator–DSE interactions in terms of activity on input devices, but only from mouse and keyboard activity [19,20]. Other quantitative studies have been performed by measuring muscle activity through procedures that interfere with workers’ jobs, such as electrogoniometers and inclinometers [21,22] or electromyography [23,24]. One study suggests behavioral sampling as an indirect technique for estimating operator–DSE interactions [25]. In the literature, we could not find systems able to collect data from screen fixation objectively and directly for a DSE operator [26,27].

The availability of objective data about active operator–DSE interaction times can allow a better characterization of the relationships between health and active DSE use, with the main aim of predisposing appropriate preventive measures. This is a true challenge since we estimate more than twenty million DSE workers in Italy (*McKinsey Global Institute*, 2017), and this will only increase due to smart-working at home as well.

We conceived, created, and patented an original and innovative system to quantitatively acquire and objectively monitor active worker–DSE interactions in a noninvasive manner without obstructing the worker’s normal activities.

This study was performed with the main aim of evaluating the ability of our system to acquire objective data of operator–DSE active interactions through screen fixation and mouse and keyboard handling. As a secondary aim, we evaluated whether and to what extent neuropsychological abilities and other factors can affect the individual mode of conducting a standardized task at a DES workstation. Finally, we evaluated the individual profiles of operator–DSE combined interactions.

## 2. Methods

**Figure 1:**
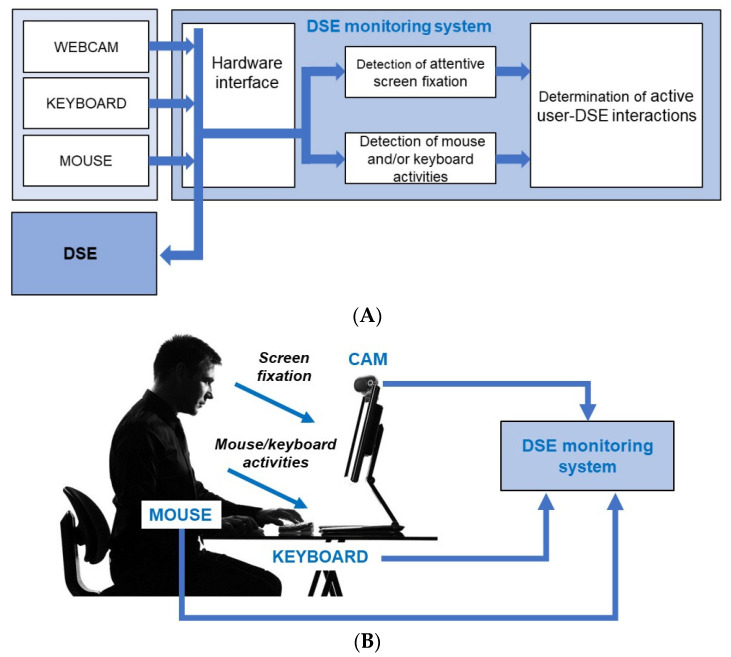
(**A**) Block diagram of the proposed system; (**B**) System collecting input data from a DSE-operator at a DSE workstation.

The block diagram of our system is shown in Figure 1A. The system is composed of a commercial webcam that can detect the worker’s attentive fixation to the monitor with eye tracking and face detection technologies (screen fixation): a hardware interface that can detect electrical inputs generated from mouse (movement, click and scroll) and/or keyboard activities; and a hardware system formed by a mini-PC hosting software for the acquisition and processing of collected data (Figure 1B).

The system is meant to work as an independent stand-alone instrument that does not interfere with the DSE operator and the software processes that are running therein. The system records the input hardware activity through the input peripherals (mouse and keyboard) and the attentive screen fixation, but it does not register any sensible content or other data that could compromise operator privacy. The system allows the detection of each single elemental event related to the use of input peripherals and screen fixation. It determines the partial usage time of single input peripherals or screen fixation by considering successive elemental events. If they are separated by less than a preset threshold window of 1 s, the activity is considered performed without interruption, and the partial usage time counter keeps running. If no elemental events occur within the threshold window, the partial usage time counter is halted waiting for the new elemental event. Moreover, the system examines the combined usage of input peripherals (mouse and keyboard) and attentive screen fixation. Specifically, three combined cases are considered: (i) usage of at least one of the input peripherals or screen fixation, (ii) simultaneous usage of all input peripherals and screen fixation, and (iii) usage of at least one of the input peripherals and simultaneous screen fixation.

We performed a cross-sectional observational study between March 2022 and July 2022 on DSE operators of the University Hospital “Spedali Civili di Brescia” and of the University of Brescia. They were enrolled as part of health promotion in the context of occupational health surveillance that is mandatory by law in Italy. Thus, the study did not need a preliminary Ethics Committee evaluation. All subjects signed an informed consent form, and the study followed the ethics principles of the Helsinki Declaration (Helsinki, 1964, Edinburgh, 2000).

The inclusion criteria were chosen according to the main characteristics of our workforce engaged as DSE operators: native Italian speaker; age from 21 to 65 years old; at least 13 years of schooling; and a negative history of neurologic or psychiatric disease.

All participants underwent additional specialized evaluations carried out by trained physicians and health personnel, including (i) eye and eyesight examination; (ii) neurological examination; and (iii) neuropsychological evaluation to exclude dyslexia and dysgraphia (via the word reading test and nonword reading test) [28,29], visual and selective auditory attention deficit (via the Test of Everyday Attention—TEA) [30] and cognitive impairment (via the Montreal Cognitive Assessment—MoCa) [31].

Subjects falling within the normal ranges of the neuropsychological evaluations were requested to complete two predefined tasks, i.e., a READ test and a COPY test, using a VDU workstation connected to the monitoring system. The READ test consisted of reading out loud a text, presented on desktop and calibrated by age, while the time to get the job done was measured via a chronometer. The COPY test consisted of copying the same text plus three other texts on the same DSE station, using Microsoft Word^®^ as the word-processing program and respecting the format of the original files. The READ and COPY texts were the same as those used to diagnose and clinically monitor dyslexia and dysgraphia. They consisted of a short excerpt from an Italian book (“*Il bar sotto il mare*” by S. Benni), a set of numbers, a set of nonwords and a set of short sentences (provided as Supplementary material).

Sociodemographic variables, as well as the results of the neuropsychological test, the time spent to perform the READ task (assessed via a chronometer) and the DSE-operator activity times collected by our system for the READ and COPY tasks were recorded in a Microsoft Excel^®^ database that was then imported into IBM-SPSS software^®^ ver. 27.0.1. for data analysis.

The Kolmogorov–Smirnov test was applied for continuous variables to evaluate their distributions and a descriptive analysis was run to calculate measures of position and dispersion. Continuous variables are presented as medians, IQR and min/max. Pearson’s correlation and linear regression analyses were then performed to evaluate the relationships between variables. A generalized linear model (GLM) analysis was finally performed to study how different independent variables (X, neuropsychological tests) could influence different dependent variables (Y, data collected through the system). Finally, we performed a sample concentration analysis through the Gini coefficient, which measures the degree of concentration (inequality) of a variable in a distribution of its elements. A two-sided α level of 0.05 was used for all tests.

## 3. Results

We enrolled 40 DSE operators, consecutively convened at the outpatient occupational health clinic for periodical health surveillance at work, with a 100% adhesion rate. Two of them were, however, subsequently excluded from data analysis because of altered neuropsychological examinations, one being diagnosed with dyslexia, and the other showing moderate attention deficit.

The remaining 38 subjects, 19 males and 19 females, showed a median age of 33.9 years (range 23–64 years). Among males, 17 (89%) were younger than 45 years, and 2 (11%) were older than 45 years; 13 (68%) females were younger than 45 years, and 6 (32%) were older than 45 years. The median schooling period was 18 years, range 13–19 years.

Table 1 presents the results of neuropsychological testing, either as a descriptive analysis of scores and as prevalence of any altered tests.

After the neuropsychological evaluation, 38 subjects completed the READ and COPY tasks, the descriptive results of which are reported in Table 2.

This shows the descriptive statistics of different operator–DSE interaction times recorded during both tasks and of errors made performing the COPY task. The session durations indicate the times between the first and the last input acquired by the monitoring system, from the camera only in the READ task and from camera or mouse or keyboard in the COPY task. All other times were calculated by the system, with time intervals ΔT = 1 s for sequential inputs from peripherals. In the READ task, times collected by the system and by the examiner were homogeneous. As expected, in the COPY task, the operators were engaged in screen fixation (median of 1261 s) more than in pressing the keyboard (median of 998 s) and again more than in using the mouse (median of 286 s). Most of the interaction times were spent combining screen fixation with mouse or keyboard use (median of 1044 s).

In the READ task, times were highly significantly related to each other (*p* < 0.0001), with r coefficients between 0.72 (between oral reading aloud and screen fixation) and 0.98 (between session duration and screen fixation).

**Figure 2:**
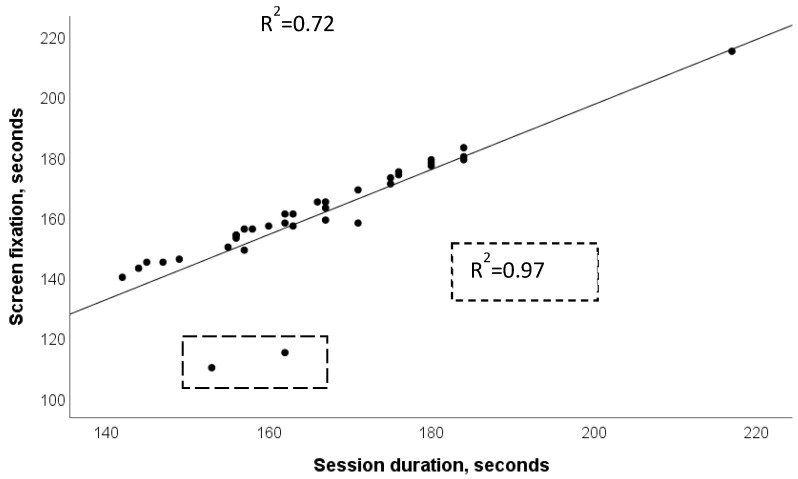
READ task. Regression line and determination coefficient between session duration and screen fixation time. Dotted boxes show the effect of the removal of two outliers from the analysis.

Figure 2 shows the regression line between session duration and screen fixation time during the READ task. There are two outliers, with very low screen fixation times compared to others. We suspected that some error could have affected such times (misplacement of the camera or of the operators during the task). Excluding such data from the analysis, the R^2^ would become 0.97.

Table 3 summarizes the results of Pearson’s correlation analysis between different activity intervals acquired by the system during the COPY task. All variables are significantly related to each other, as expected, with the highest values being represented by the relationships between session duration and inputs from at least one of the peripherals or screen fixation (r = 0.97, *p* < 0.0001). The session duration was strongly related to mouse activity (r = 0.93, *p* < 0.0001), followed by keyboard activity (r = 0.90, *p* < 0.0001) and screen fixation (r = 0.52, *p* < 0.005). The screen fixation was similarly related to mouse (r = 0.46, *p* < 0.005) and keyboard (r = 0.48, *p* < 0.005) activities.

The number of mistakes during the COPY task was unrelated to any of the activity times.

**Figure 3:**
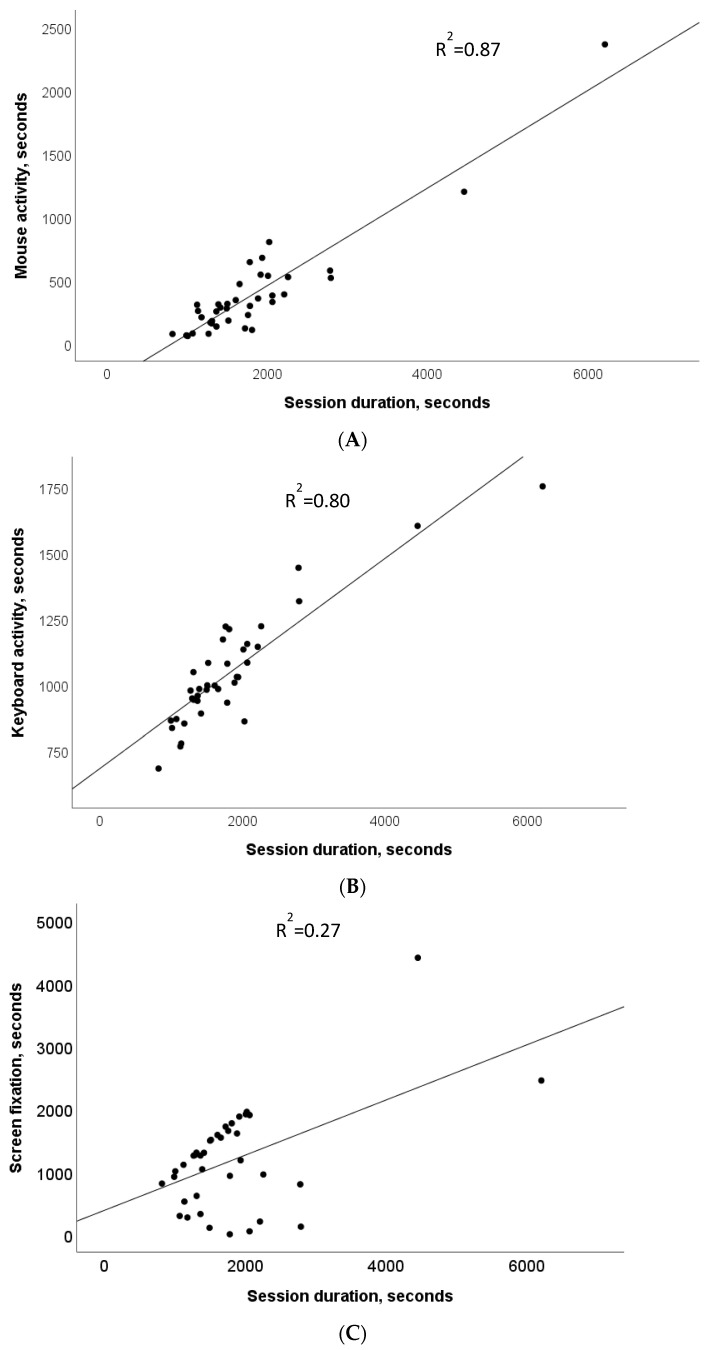
COPY task. Regressions lines and determination coefficients between session duration and activity times from different peripherals, mouse (**A**), keyboard (**B**) and screen fixation (**C**).

Figure 3 shows the regressions between session durations of the COPY task and times of active interaction with different peripherals or screen fixation by different operators. Mouse activity showed the highest determination coefficient (R^2^ = 0.87, *p* < 0.0001), and screen fixation showed the lowest (R^2^ = 0.27, *p* < 0.001).

Variables, such as age, gender, dominant hand, and schooling, showed no significant relationship with any of the operator–DSE interaction times acquired during the READ/COPY tasks.

Then, we evaluated whether and how the results of the neuropsychological test could influence operator–DSE interaction times using a generalized linear model (GLM). We found four statistically significant relationships in the COPY task: three regarding TEA for visual attention with session duration (*p* = 0.010), mouse activity (*p* = 0.009), and combined activity (*p* = 0.006) and one between error of omission in TEA and number of errors made (*p* = 0.003) (Supplementary Material, Table S1). Such associations showed statistical powers between 0.76 and 0.89. Linear regression analyses showed that the relationships were all positive. Such results were implemented via a concentration analysis, constructing the Gini index for four classes of values of TEA; the Gini index reaches a value equal to 5.7% of its theoretical maximum, thus highlighting a low concentration of TEA scores in the sample (Figure S1).

**Figure 4:**
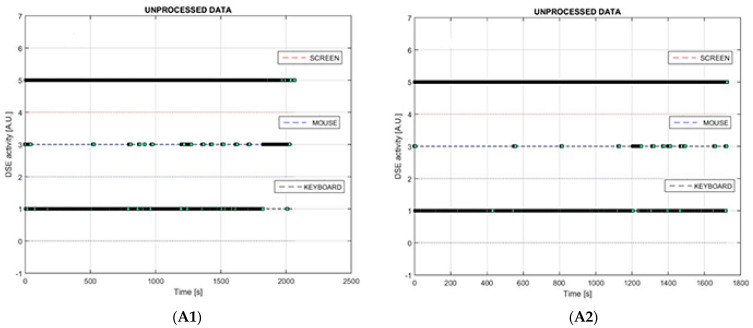

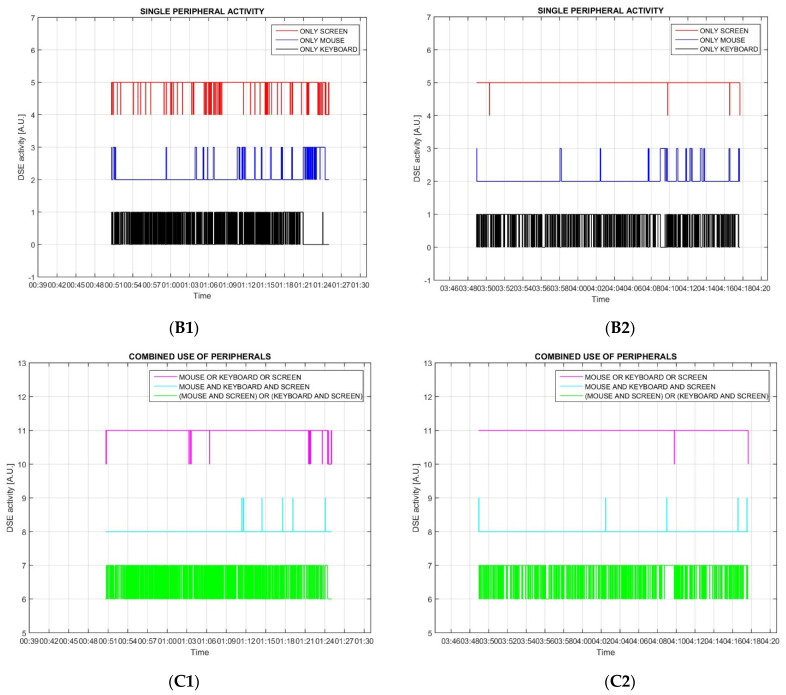
In each graph, the x-axis is a time scale (seconds). (**A1**,**A2**) show the sequence of unprocessed inputs for every peripheral; (**B1**,**B2**) show activities from mouse (blue line), keyboard (black line) or screen fixation (red line). Each spike corresponds to an input activity, whereas the horizontal lines indicate lack of activity; (**C1**,**C2**) show the combined use of peripherals according to the color legend, spikes correspond to combined activity, while the horizontal lines indicate lack of combined activity.

Figure 4 shows some examples of graphs of operator–DSE interactions that can be produced by the monitoring system. In this case, graphs refer to two operators (−1 and −2) performing the COPY task. In each graph, the x-axis is a time scale (seconds). The unprocessed data graphs (Figure 4(A1,A2)) show the raw sequence of inputs from each input peripheral and each event of screen fixation.

In the graphs describing the activities of any single input peripheral or single screen fixation with the relative partial usage time (Figure 4(B1,B2)), each high-level spike corresponds to a single activity, whereas the low-level horizontal lines indicate lack of activity. (Figure 4(C1,C2)) show combined peripheral activity and screen fixation times, again, high-level spikes corresponding to combined activities, while the horizontal low-level lines indicate lack of combined activity.

From the profiles of Figure 4, we can evaluate that operator 2 could copy the text without almost never watching the screen; he took less time copying a text but with more errors than operator 1, who took more time, with fewer errors and watched the screen more often. The obtained patterns obviously depend on the task that operators are requested to complete.

## 4. Discussion

Occupational exposure to DSE is bound to increase rapidly, because of the digital revolution in the work world (*McKinsey Global Institute*, 2017). However, the identification of at-occupational risk DSE operators is not based on completely objective criteria, in most cases relating occupational risk to the time actively spent at DSE workstations, which is collected through self-administered questionnaires or checklist guided interviews. Such a mode of risk assessment is prone to main critical biases (e.g., evident discretion, poor accuracy in estimating usage time of mouse or keyboard, and poor objectivity) which can lead either to overestimation or underestimation of the risk [32], whereas the design and implementation of appropriate and effective prevention measures should rely on accurate and reliable quantitative high-quality risk assessment.

The need to have objective, specific and complete data on DSE operators’ activity was conducive to the collaboration leading our university groups made of occupational health physicians and information engineers to conceive, develop, and patent a new system able to collect data from the active interaction between DSE operators and PC peripherals and screen fixation. 

The system we developed can collect every active interaction of the operator with key DSE components, i.e., screen, keyboard, and mouse. Elementary input data can then be integrated and analyzed to obtain either times of active interactions with peripherals or operator–DSE interaction profiles.

To evaluate the system, we designed a semiexperimental cross-sectional study, consecutively enrolling a group of healthy DSE operators with similar experience in DSE use, and after excluding two of them due to detecting neuropsychological issues (diagnosis of dyslexia and moderate attention deficit), the group could be considered almost homogeneous for the study purposes. Subjects performed two standardized tasks, the first evaluating the ability of the system to collect screen fixation times and the second evaluating the times of active interaction with screen, keyboard, and mouse. The obtained results indicate good data coherence between different activity times as monitored by the system. In the READ task, screen fixation showed a good relationship with session duration, whereas in the COPY task, the latter showed the best relationship with mouse activity. When looking at possible relationships among neuropsychological tests and descriptors of operator–DSE interactions, we found statistically significant associations between TEA for visual attention score and session duration time, mouse activity and any single peripheral activity. All such relationships were positive, i.e., the higher the TEA visual attention score, the higher the time required to copy the text actively interacting with the DSE. In addition, we also found a statistically significant association between the TEA errors of omission score and errors in the copied text, also in this case positive, i.e., the higher the TEA error score, the higher the number of mistakes. We failed to find other possible factors affecting the collected data among age, gender, dominant hand and schooling, but the obtained results should be judged with caution, owing to the small sample size that we studied.

Finally, the proposed system can integrate elementary and instant operator–DSE interactions, allowing the definition of individual interaction profiles, which can be noticeably different from each other. The temporal sequences of activities, the distributions of the relative partial interaction times with different peripherals times, and the temporal distributions of combined activities can allow us to obtain information about different styles, dexterity levels and approaches in using DSE, typical of the operator, as well as to detect periods of inactivity at DSE of operators in DSE workstations.

Our monitoring system is an innovative tool, and, compared with similar systems on the market in Italy (e.g., DSE-Meter by *Provincia Autonoma di Trento*, *Italy* or wearable devices) [33,34] or previously described in the literature [19,20], it allows objective measurement of visual and musculoskeletal system strain, distinguishing the use time of a single peripheral, the use time of peripherals in combination and the total working time. To the best of our knowledge, this is the first system to be patented with such characteristics. The importance of obtaining objective data would allow recording the time of activity to DSE, making it possible to objectify risk related to screen fixation, mouse–keyboard use or overall engagement at the workstation. Times of active interactions with DSEs can thus be used (i) to stratify workers in bands of risk and (ii) to introduce work breaks to manage risk, particularly in vulnerable workers. Individual operator–DSE profiles can be evaluated to correct individual behaviors leading to anomalous excess of visual (screen fixation) or hand engagement, particularly for mouse activity.

Data from the proposed monitoring system allow a more thorough and objective analysis of the relationships, if there is any between visual activity and MSD in DSE operators, and helps in implementing the most appropriate prevention and protection measures. The monitoring system not only identifies the operator’s presence but specifically detects the intentional attentive gaze at the screen, demonstrating active interaction; on the other hand, the system ensures that worker privacy is respected.

The international scientific literature has long highlighted associations between MSD outcomes and posture and intensity of keyboard use (hours of computer use per day or week) by DSE operators with associations most pronounced for timing and frequency [8]. On the other hand, the low quality of information about time actively spent at the DSE workstation hinders the risk assessment of biomechanical overload to the musculoskeletal system since this requires reliable exposure times. Such information is also pivotal to assess the role of work in the genesis of such disorders and to identify hypersusceptible individuals, given the wide prevalence of musculoskeletal disorders and diseases in the general population not actively working with DSEs. With reference to the hand-wrist anatomy, for instance an epidemiological study found that symptoms in this region are common in the general population: the prevalence in men and women is 1.1% and 2.2%, respectively. In contrast, nonspecific pain of the wrist and hand is much more common: 8.7% in men and 11.5% in women [35]. Pictures of distal tendinopathies have been described in the tendons of the flexor and extensor muscles of the fingers and carpus in both occupational and athletic settings [36]. Recently, the use of smartphones has also been considered a risk factor for the occurrence of hand tendinitis [37]. When referring to shoulder disorders, painful shoulder constitutes a common MSD, with prevalence ranging from 16% to 26%. More than 60% of patients presenting with a painful shoulder may have symptoms for more than a year [38]. In the U.S., 20% of the population experiences shoulder pain during their lifetime, and this disorder is second only to low back pain among musculoskeletal disorders presenting to the general practitioner [39]. With reference to cervical spine, another region particularly strained by VDU use, data on cervicalgia show that neck pain is a serious public health problem in the general population. Increased population awareness of risk factors and preventive strategies for neck pain is warranted to reduce the future burden of this condition [40].

Strength. The strengths of our work rely on the experimental study design of using standardized tasks and on the preliminary visual and neuropsychological assessment of participants allowing a control of variables potentially affecting the operator–DSE interactions.

Limitations. A main study limitation was the small number and a certain homogeneity of the sample. However, the small number did not affect the statistical power of the observed findings (Table S1). Regarding the second issue, through the Gini test, we could verify that at a statistical level, the sample was not as homogeneous as in appearance. We need to collect more data, expand the sample, and introduce new variables (e.g., schooling less than 13 years, positive history of musculoskeletal and neurological diseases) to better evaluate the potential of the system.

Future research directions. The next experimental step will be the validation of the system, which could require videorecording of the DSE operators in parallel with the acquisition of data by the system.

Data collected by the system can also be useful not only for health but also for organizational and managerial purposes in occupational settings where the operator–DSE interaction is the main professional content (banking, finance, journalism, graphics, etc.). For instance, the evaluation of time actively spent at DSE workstations can support the analysis of the commitment required for the workforce by adopting new software, which can be evaluated in terms of cost–benefit analysis. Similarly, the same data can be used to evaluate the effectiveness of educational actions on DSE operators.

With the aim of guaranteeing the highest level of wellbeing in DSE operators, data collected by the system may support analyses of accessibility and usability of the enterprise software, which may play a role in mental workload. This holds particularly useful in sectors where software is frequently changed.

Analyzing the data obtained by the system, we can study individual ways of interacting with DSEs, identifying each subject, as if they were unique fingerprints. Extremely interesting from a neuropsychological point of view is the possibility of identifying and monitoring individual profiles of operator–DSE interactions over time. From the perspective of progressive improvement in the ability to interact with DSEs as a result of training, education, and experience, the profiles should demonstrate interactions that are always more favorable from a risk perspective. On the other hand, a promising research field is the study of the interaction with digital devices in neurodegenerative pathologies, such as Parkinson’s disease [41] or multiple sclerosis [42]. In fact, according to new research data, tap speed (how quickly one types on a smartphone keyboard) may be a useful tool for monitoring multiple sclerosis severity [43,44]. Thus, we cannot exclude the application of the prospective analysis of operator–VDU interaction with our system to promote early diagnosis of neurodegenerative alterations. A recent study revealed how typing speed in combination with depression scores has the potential to infer aspects of cognition, such as visual attention, and are useful to complement neuropsychological assessments [45].

Our system is easily transportable, and thus it is possible to use for smart-working employees as well. The risk assessment of DSE operators working at home would be a new and interesting field of application for our system.

## 5. Conclusions

In conclusion, the results obtained, although preliminary due to the small sample size examined, demonstrate that our system is capable of tracking active operator–DSE interactions at work through screen fixation and peripheral use (mouse, and keyboard). Further studies are needed to assess the validity and reliability of the data obtained, which can ideally be used for various purposes in occupational settings related to the health, wellbeing, training, and productivity of DSE operators. We consider our system to be in a premarketing and experimental stage, which needs further validation before thinking about using it extensively in the work environment. The system can be produced with commercially available, consumer-grade electronic components, so its final production cost is expected to be compatible with widespread acceptance and use in workplaces.

## Figures and Tables

**Table 1:** Distributions of the results of neuropsychological tests in our study sample (*N* = 40).

Neuropsychological Test	Median	Min–Max	Out of Range Values (%)
*MoCa*. Score	27	23–30	1/40 (2.5%)
*TEA for selective auditory attention*, reaction time, s	654	446–806	8/40 (20%)
*TEA for visual attention*, reaction time, s	786	659–1009	0/40
*TEA*, error of commission, n°	1	0–14	4/40 (10%)
*TEA*, Error of omission, n°	1	0–8	4/40(10%)
*Word reading test* duration, s	59	44–109	4/40 (10%)
*Word reading test*, error of commission, n°	1	0–3	0/40
*Nonword reading* test, s	53	36–97	4/40 (10%)
*Nonword reading test*, errors of commission, n°	1	0–6	1/40 (2.5%)

**Table 2:** Descriptive statistics of variables collected by the system (*sy*) or by examiners (*ex*, *by chronometer*) during the READ and COPY tasks on 38 enrolled subjects.

Tasks/Parameters	Median (IQR)	Min–Max
READ		
Session duration, *s*, *sy*	163 (156–176)	142–217
Oral reading aloud, *s*, *ex*	147 (142–154)	134–206
Screen fixation, *s*, *sy*	159 (151–174)	110–215
COPY		
Session duration, *s*, *sy*	1518 (1302.5–1930)	821–6212
Screen fixation (1), *s*, *sy*	1261 (868–1633)	108–4413
Mouse activity (2), *s*, *sy*	286 (164–431)	61–2367
Keyboard activity (3), *s*, *sy*	998 (916–1110)	684–1754
(1) OR (2)OR (3), *s*, *sy*	1539 (1246–1837)	819–4868
(1) & (2) OR (1) & (3), *s*, *sy*	1044 (512.2–1287)	2–2722
(1) & (2) & (3), *s*, *sy*	15.5 (7–31)	0–70
Copying, errors (incorrectly copied words), no, *ex*	9 (5–20)	2–50

IQR: interquartile range, 25th–75th percentiles.

**Table 3:** Results of Pearson’s correlation analysis between operator–DSE activity times through different peripherals, as collected by the system during the COPY task.

COPY Task, Activity Times, s	Session Duration	Screen Fixation (1)	Mouse Activity (2)	Keyboard Activity (3)	(1) OR (2) OR (3)	(1) & (2) & (3)
Screen fixation (1)	0.52 ***					
Mouse activity (2)	0.93 ***	0.46 *				
Keyboard activity (3)	0.90 ***	0.48 *	0.70 ***			
(1) OR (2) OR (3)	0.97 ***	0.70 ***	0.90 ***	0.87 ***		
(1) & (2) & (3)	0.50 ***	0.67 ***	0.58 ***	0.39 *	0.60 ***	
(1) & (2) OR (1) & (3)	0.37 *	0.97 ***	0.36 *	0.34 *	00.57 ***	0.67 ***

* *p* ≤ 0.050; *** *p* ≤ 0.001.

## Data Availability

The authors are responsible for the correctness of the data and can share with other researchers the anonymized data set including all the data used on request by qualified investigator.

## Supplementary Materials

The following supporting information can be downloaded at: https://www.mdpi.com/article/10.3390/bioengineering10060686/s1, Figure S1: Gini graph of TEA visual attention, showing the cumulative relative frequencies on the abscissa axis and the cumulative relative quantities on the ordinate axis; the area under the equidistributional line expresses the area of maximum concentration; Table S1: Main results of general linear model analysis between neuropsychological test scores and outcome variables. Gray boxes represent outcomes collected in the COPY task; white boxes are referred to outcomes collected during the READ task.
