# Supplementary material for: Objective Evaluation of Active Interactions between the Operator and Display Screen Equipment Using an Innovative Acquisition System
Source: Bioengineering (Basel). 2023 Jun 4;10(6):686. doi: 10.3390/bioengineering10060686 (PMC10295591; doi:10.3390/bioengineering10060686)

Supplementary Materials

Text used in the READ and COPY tasks

**a) Il bar sotto il mare**
Non so se mi crederete. Passiamo metà della vita a deridere ciò in cui altri credono, e l'altra metà in ciò che altri deridono. Camminavo una notte in riva al mare di Brigantes, dove le case sembrano navi affondate, immerse nella nebbia e nei vapori marini, e il vento dà ai rami degli oleandri lente movenze di alga. Non so dire se cercassi qualcosa, o se fossi inseguito: ricordo che erano tempi difficili ma io ero, per qualche strana ragione, felice.<br>Improvvisamente dal sipario del buio uscì un vecchio elegante, vestito di nero, con una gardenia all'occhiello, e passandomi vicino si inchinò leggermente. Mi misi a seguirlo incuriosito. Andavo di buon passo ma faticavo a stargli dietro, perché sembrava che procedesse volando a un palmo da terra, e i suoi piedi non facevano rumore sul legno umido del molo. Il vecchio si fermò un attimo, tracciando in aria gesti con cui sembrava calcolare la posizione delle stelle. Poi annuì con la testa e prese a discendere una scaletta che dal molo calava nelle acque scure. - Si fermi signore – gridai – non lo faccia! Ma il vecchio non mi ascoltò, in breve tempo fu nell'acqua fino alla cintola, e poco dopo scomparve.<br>Senza indugiare, vestito com'ero, mi tuffai. L'acqua era gelida, e sul fondale melmoso giacevano detriti e cordami. Mi guardai intorno cercando tracce dell'uomo e con mia grande meraviglia vidi, sospesa a pochi metri dal fondo, un'insegna luminosa con la scritta "Bar". Verso di essa si dirigeva tranquillamente, camminando come un palombaro, il vecchio della gardenia. Come in un sogno nuotai anch'io verso quell'insegna che illuminava l'acqua di azzurro. Arrivai così a una costruzione intarsiata di nautili, con una porta di legno.<br>La porta si aprì subito e il signore con la gardenia mi tese la mano. Non fece altro che tirarmi dentro di colpo e mi trovai in un bar accogliente, luminoso e pieno di avventori.<br>Era arredato con mobili di stile diverso, alcuni di antico gusto marinaro, altri esotici, altri decisamente moderni. Il bancone sembrava la fiancata di una nave, tanto era lucido e imponente. Sopra lo schieramento delle bottiglie c'era un grande oblò di vetro da cui si potevano ammirare candelabri di corallo e branchi di pesci. Gli avventori bevevano e chiacchieravano come in un qualsiasi bar di terraferma.<br>Come potete constatare dal disegno di copertina, formavano il gruppo più stravagante che io avessi mai visto. Il barista mi fece segno di avvicinarmi. Aveva un'espressione ironica e il suo volto ricordava quello di un famoso interprete di fil dell'orrore.

Mi offrì un bicchiere di vino e mi appuntò una gardenia all'occhiello. - Siamo lieti di averla tra noi – disse sottovoce. La prego di accomodarsi, perché, questa è la notte in cui ognuno dei presenti racconterà una storia. Mi sedetti, e ascoltai i racconti del bar sotto il mare.
(tratto da S. Benni, *Il bar sotto il mare*, © Giangiacomo Feltrinelli Editori, Milano, 1998)

Additional texts used in the COPY task

b) **Set of nonword**	
Spato	Badapana
Chiampa	Dopustrigli
Cogheste	Chicheporu
Tatripsi	Putrignu
Manumolli	Mamelestu
Tigiglione	Sirfonuglio
Riumtaghire	Scefo
Strascridi	Parda
Clabiturra	Trugnusco
Pavalatto	Senamirca

**c) Set of Numbers**	
2659452526	4174769183
4125130491	2742629450
0676898080	4340430282
9043019376	6156158369
7953788976	4528139151
7376858532	7975073408
5231231728	4174769183
2742629450	

**d) Set of short sentences**
Davide è canadese e ha 19 anni.
Trascorre le vacanze in Francia.
Fa visita al suo amico Moris.
Hanno visitato Parigi e hanno visto la Tour Eiffel. Questa sera vanno al parco giochi con la metro. C'è tanta gente e rumore.
Vedono le giostre.
Davide intravede da lontano un borseggiatore.
Il borseggiatore fruga nella borsa di una donna vestita di rosso.
Davide interpella il borseggiatore.<br>Tutti i passanti si girano e il borseggiatore scappa.
La donna vestita di rosso ringrazia Davide e Moris e offre ai ragazzi un giro sulla panoramica.

Table S1. Main results of general linear model analysis between neuropsychological test scores and outcome variables. Gray boxes represent outcomes collected in the COPY task; white boxes are referred to outcomes collected during the READ task.

ORIGIN	DEPENDENT VARIABLE	TYPE III SUM OF SQUARES	QUADRATIC MEAN	F	P	OBSERVED POWER[K]
**TEA for visual attention**	Session duration	6698981.492	6698981.492	7.764	0.010	0.762
	Screen fixation	1741714.258	1741714.258	2.604	0.120	0.341
	Mouse activity	1266054.652	1266054.652	8.011	0.009	0.775
	Keyboard activity	155325.108	155325.108	3.717	0.066	0.457
	Any single peripheral active	5096639.545	5096639.545	8.900	0.006	0.816
	Three peripherals active	609.052	609.052	1.676	0.208	0.237
	Screen fixation & (mouse or keyboard)	282097.048	282097.048	0.855	0.364	0.144
	Copying error of commission	178.328	178.328	1.227	0.279	0.186
	Session duration	345.934	345.934	1.687	0.206	0.239
	Screen fixation	1143.417	1143.417	3.495	0.074	0.434
**TEA error of omission**	Session duration	842042.507	842042.507	0.976	0.333	0.158
	Screen fixation	146594.415	146594.415	0.219	0.644	0.073
	Mouse activity	178265.114	178265.114	1.128	0.299	0.175
	Keyboard activity	33983.883	33983.883	0.813	0.376	0.139
	Any single peripheral active	510269.322	510269.322	0.891	0.355	0.148
	Three peripherals active	261.883	261.883	0.721	0.404	0.129
	Screen fixation & (mouse or keyboard)	63043.616	63043.616	0.191	0.666	0.070
	Copying error of commission	1589.219	1589.219	10.939	0.003	0.887
	Session duration	64.817	64.817	0.316	0.579	0.084
	Screen fixation	5.569	5.569	0.017	0.897	0.052

Table 2 [k] α =.05

Figure S1. Gini graph of TEA visual attention, showing the cumulative relative frequencies on the abscissa axis and the cumulative relative quantities on the ordinate axis; the area under the equidistributional line expresses the area of maximum concentration.

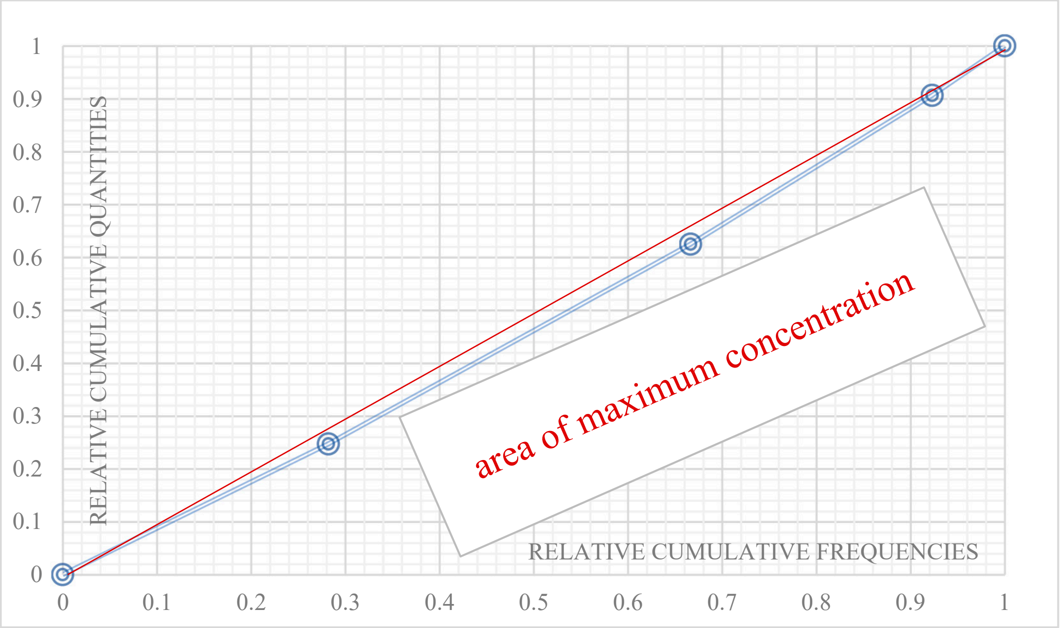

Supplement: Supplementary file 1 [file bioengineering-10-00686-s001.zip › bioengineering-2422164-supplementary.pdf]
